# Identification and characterization of plasmids carrying the mobile colistin resistance gene *mcr-1* using optical DNA mapping

**DOI:** 10.1093/jacamr/dlad004

**Published:** 2023-02-01

**Authors:** Sriram KK, Moa S Wranne, Tsegaye Sewunet, Elina Ekedahl, Maarten Coorens, Teerawit Tangkoskul, Visanu Thamlikitkul, Christian G Giske, Fredrik Westerlund

**Affiliations:** Department of Biology and Biological Engineering, Chalmers University of Technology, Gothenburg, Sweden; Department of Biology and Biological Engineering, Chalmers University of Technology, Gothenburg, Sweden; Division of Clinical Microbiology, Department of Laboratory Medicine, Karolinska Institute, Stockholm, Sweden; Department of Biology and Biological Engineering, Chalmers University of Technology, Gothenburg, Sweden; Clinical Microbiology, Karolinska University Hospital, Stockholm, Sweden; Faculty of Medicine Siriraj Hospital, Mahidol University, Bangkok, Thailand; Faculty of Medicine Siriraj Hospital, Mahidol University, Bangkok, Thailand; Division of Clinical Microbiology, Department of Laboratory Medicine, Karolinska Institute, Stockholm, Sweden; Clinical Microbiology, Karolinska University Hospital, Stockholm, Sweden; Department of Biology and Biological Engineering, Chalmers University of Technology, Gothenburg, Sweden

## Abstract

**Objectives:**

Colistin is a last-resort antibiotic, but there has been a rapid increase in colistin resistance, threatening its use in the treatment of infections with carbapenem-resistant *Enterobacterales* (CRE). Plasmid-mediated colistin resistance, in particular the *mcr-1* gene, has been identified and WGS is the go-to method in identifying plasmids carrying *mcr-1* genes. The goal of this study is to demonstrate the use of optical DNA mapping (ODM), a fast, efficient and amplification-free technique, to characterize plasmids carrying *mcr-1*.

**Methods:**

ODM is a single-molecule technique, which we have demonstrated can be used for identifying plasmids harbouring antibiotic resistance genes. We here applied the technique to plasmids isolated from 12 clinical *Enterobacterales* isolates from patients at a major hospital in Thailand and verified our results using Nanopore long-read sequencing.

**Results:**

We successfully identified plasmids encoding the *mcr-1* gene and, for the first time, demonstrated the ability of ODM to identify resistance gene sites in small (∼30 kb) plasmids. We further identified *bla*_CTX-M_ genes in different plasmids than the ones encoding *mcr-1* in three of the isolates studied. Finally, we propose a cut-and-stretch assay, based on similar principles, but performed using surface-functionalized cover slips for DNA immobilization and an inexpensive microscope with basic functionalities, to identify the *mcr-1* gene in a plasmid sample.

**Conclusions:**

Both ODM and the cut-and-stretch assay developed could be very useful in identifying plasmids encoding antibiotic resistance in hospitals and healthcare facilities. The cut-and-stretch assay is particularly useful in low- and middle-income countries, where existing techniques are limited.

## Introduction

There is a growing concern in tackling antimicrobial resistance (AMR), which has developed into a main threat to global health.^1^ Existing antibiotics are becoming obsolete in treating patients and there is an urgent need for antimicrobials with novel mechanisms of action. Moreover, it is important to develop methods to identify and characterize bacteria carrying significant resistance genes.

Mobile genetic elements, such as plasmids, transposons, and to some extent bacteriophages, play a major role in the spread of AMR. Among them, resistance genes located on plasmids, extrachromosomal DNA in bacteria that can replicate independently of the chromosome, have been studied extensively and their role in AMR has been well documented.^2,3^ Plasmids can spread between different species of bacteria via conjugation and thereby transfer resistance genes from one bacterium to the other. Importantly, several resistance genes can be located on the same plasmid.

*Enterobacterales* are important pathogens in healthcare-associated infections, and the spread of carbapenem-resistant *Enterobacterales* (CRE) is a major threat to currently used treatment regimens. Polymyxins, including colistin, have been considered last-resort antibiotics,^4,5^ and are still highly important for treatment in large parts of the world. The spread of the mobile colistin resistance-1 (*mcr-1*) gene dates back to the 1980s, as evident through retrospective investigations performed on isolates collected between the 1970s and 2014 in China.^6^ However, the first study showing plasmid-mediated resistance to colistin via the *mcr-1* gene was reported from China as late as 2015.^7^ Since then, the *mcr-1* gene, along with other *mcr* gene variants, has been reported from many different parts of the world.^8–16^*Enterobacterales* harbouring *mcr* genes have been frequently reported,^17,18^ and the rapid increase in reports of *Escherichia coli* and *Klebsiella pneumoniae* harbouring *mcr* genes reflects the magnitude of the problem.^9,19–23^

AMR in the Southeast Asian (SEA) region is of great concern and the WHO has listed SEA as likely the most affected region.^24^*E. coli* and *K. pneumoniae* harbouring *mcr* genes have been reported frequently in Thailand over the past few years.^9,25–27^ WGS has been the go-to method in such studies and although there is a major development in this area with the use of benchtop sequencers and advanced analysis tools to speed up the process, there is still a need for other methods to characterize plasmids encoding AMR genes. In particular, methods that can provide long-range sequence information of plasmids, reduce analysis time and cost and have the potential to be implemented in hospital settings in low- and middle-income countries, are desirable.

**Figure 1.**
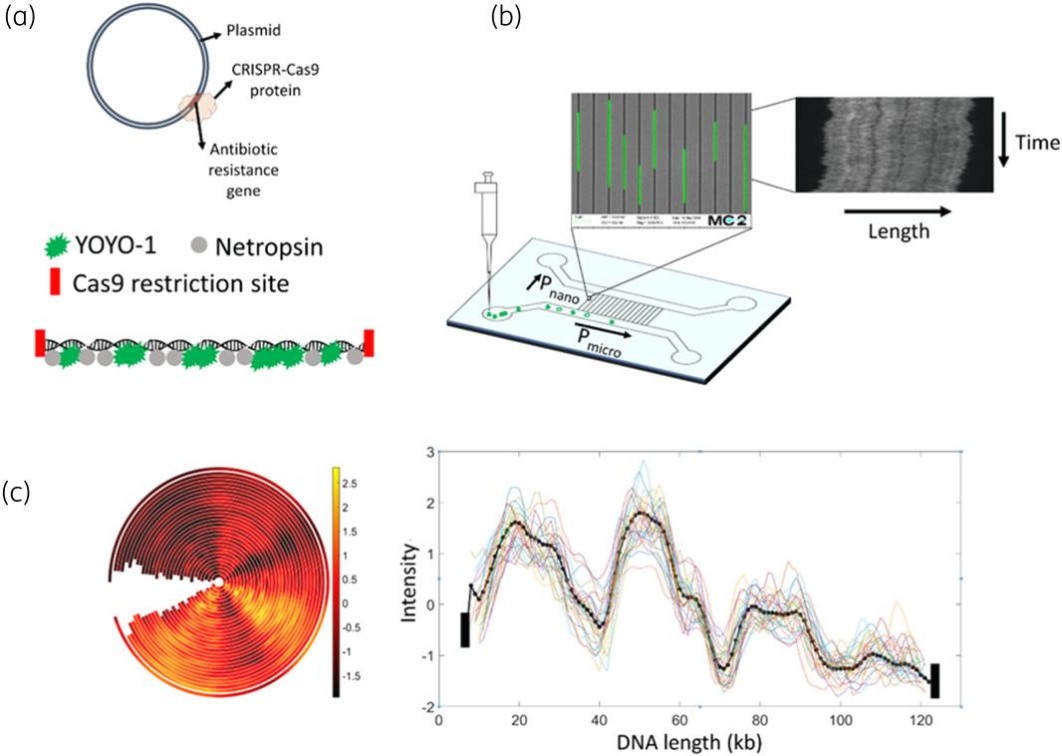
Schematics of ODM. (a) CRISPR-Cas9-based targeted restriction of antibiotic resistance gene site in a plasmid, followed by single-step YOYO-netropsin labelling. (b) DNA sample is added to the loading reservoir of the nanofluidic device and pushed into the nanochannels using pressure-driven flow. Fluorescence imaging of single DNA molecules in nanochannels is then used to obtain kymographs. Inset shows DNA molecules stretched in nanochannels. (c) Concentric plot and intensity versus length plot from tens of individual plasmids (thin lines) to obtain the consensus intensity profile (barcode, dotted thick line) and identify the location of the resistance gene. The consensus intensity profile is aligned with the Cas9-restricted resistance gene site at the ends, represented by black rectangles.

We have developed a method based on optical DNA mapping (ODM) and CRISPR/Cas9 technology to characterize plasmids on the single-plasmid level.^28–30^ The method provides the number of different plasmids in a sample, their sizes and on which plasmid a specific gene is located, as well as a ‘DNA barcode’ that can be used for characterization of the plasmids.^31–33^ Identification of the AMR gene of interest is carried out using CRISPR/Cas9, with the help of a guide RNA (gRNA) designed to target and restrict a 20 bp sequence in the resistance gene.^34^ The DNA barcode is formed by staining the DNA with two molecules, YOYO-1, which is fluorescent, and netropsin, a non-fluorescent molecule that blocks YOYO-1 from binding to AT-rich regions.^31,32^ This leads to an intensity variation along the DNA that reflects the underlying sequence and that can be used as a barcode. To reveal the barcode, the DNA is stretched in nanofluidic channels (Figure 1).^35,36^ We have used this method to identify plasmids from sequence databases and to trace plasmids during resistance outbreaks.^29,30,37,38^ We have recently also presented a simplified version of the assay that detects resistance genes on plasmids using a microscope (Primo Star iLED) that is already present in laboratories in low- and middle-income countries for diagnosing tuberculosis.^39^

In this study we demonstrated that ODM can readily identify plasmids that carry the *mcr-1* gene and that we can determine if the *mcr-1* gene is located on the same plasmid as other AMR genes, which is important from an epidemiological perspective. We finally show that plasmids carrying *mcr-1* can be identified using the cut-and-stretch microscope assay, which opens possibilities for even broader use, particularly in low- and middle-income countries.

## Materials and methods

The DNA extraction for sequencing was performed according to standard protocols as outlined in detail in the Supplementary Methods, available as Supplementary data at *JAC-AMR* Online). Illumina HiSeq 2500 and MinION Nanopore long-read sequencing data were obtained, as discussed in the Supplementary Methods, and submitted to NCBI (BioProject ID: PRJNA850449).

Plasmids were extracted using a NucleoBond^®^ Xtra Midi Kit (Macherey-Nagel) plasmid purification protocol as described in the Supplementary Methods.

The ODM protocol is schematically shown in Figure 1 and details can be found in the Supplementary Methods, along with details of the data analysis. We use a P-value (P) and a cross-correlation score (CC) to compare barcodes.

ODM was performed on 12 isolates encoding the *mcr-1* gene, of which 10 were *E. coli* and 2 were *K. pneumoniae*. Among them, short-read sequencing was performed for four isolates (T2F_2, T3F_1, T6F_2, T7F_2) and long-read Nanopore sequencing was performed for the remaining eight isolates. The isolates were named with patient numbers T1 to T8, the sample type (F = faeces; S = sputum; X = undefined) and, if more than one isolate was collected from a patient, they were listed in numerical order (_1 and _2).

The cut-and-stretch assay was performed as previously described,^39^ with details and exceptions in the Supplementary Methods.

## Results and discussion

### ODM

**Figure 2.**
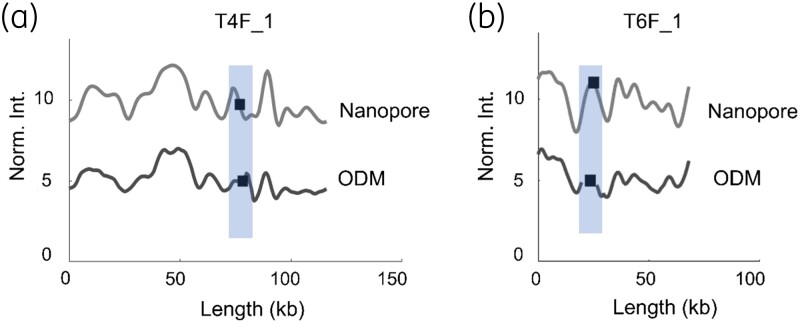
Comparison between barcode from long-read Nanopore sequencing (grey) and ODM experimental barcodes (black) for isolates (a) T4F_1 (116 kb) and (b) T6F_1 (68 kb). The *x*-axis is the length of the plasmids in kb and the *y*-axis is the normalized intensity in arbitrary units, where the intensity plots are shifted five units vertically for clarity. Black squares within the shaded regions represent the location of the *mcr-1* gene.

To demonstrate the ability of ODM to provide sequence information and the location of the *mcr-1* gene in plasmids with high precision, we compared the barcodes obtained from the ODM experiments to barcodes generated from the sequences obtained through long-read Nanopore sequencing (referred to as ‘Nanopore’).^40^ Figure 2 shows such results for two isolates. ODM suggests that isolate T4F_1 carries the *mcr-1* gene in a 116 kb-long plasmid and comparison with the Nanopore barcode showed a very good match both for the barcode (*P* < 0.01) and the *mcr-1* gene location [Figure 2(a)]. Similarly, ODM suggests that isolate T6F_1 carries the *mcr-1* gene in a 68 kb-long plasmid and, once again, there was a good match with the Nanopore barcode (*P* < 0.01), and the *mcr-1* gene location was identical [Figure 2(b)].

One interesting observation during this study was that the *mcr-1* gene in many isolates was located on smaller plasmids, in the order of 30 kb. This size is at the lower limit where ODM can provide a variation in the intensity along the DNA that can be used for analysis. We would like to stress that single-molecule DNA imaging methods can provide length information at much smaller length scales,^41,42^ but successful identification of a specific plasmid and the location of the gene of interest in the plasmid requires distinguishable intensity variations along the DNA.

**Figure 3.**
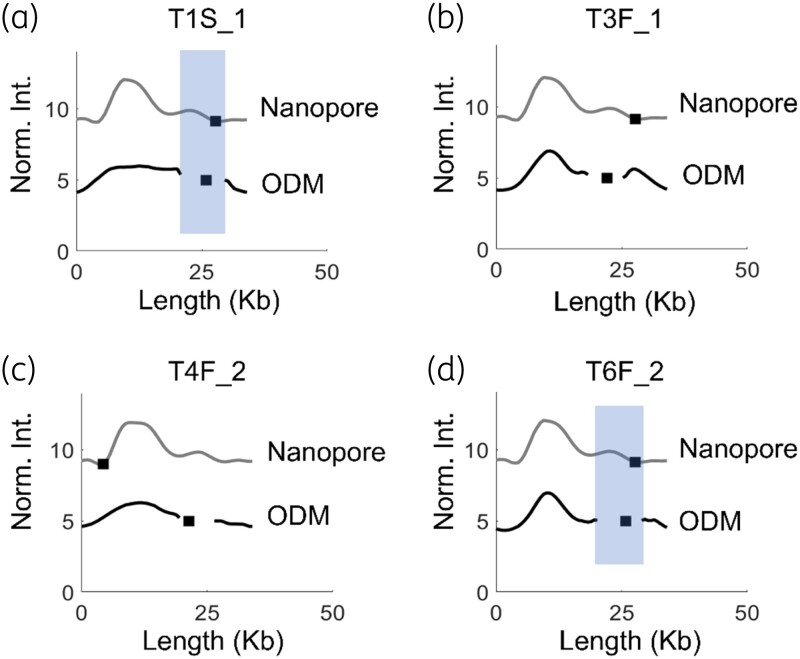
ODM barcodes of 34 kb plasmids with the *mcr-1* gene (black) compared with barcodes from Nanopore sequencing (grey) for sample T1S_1 (a), T3F_1 (b), T4F_2 (c) and T6F_2 (d). For T3F_1 and T6F_2, the Nanopore barcode is for T1S_1. The *x*-axis shows the length of plasmids in kb and the *y*-axis is the normalized intensity in arbitrary units, where the intensity plots are shifted five units vertically for clarity. Black squares represent the location of the *mcr-1* gene and the shaded regions indicate when the gene location is similar for the two tecniques.

**Figure 4.**
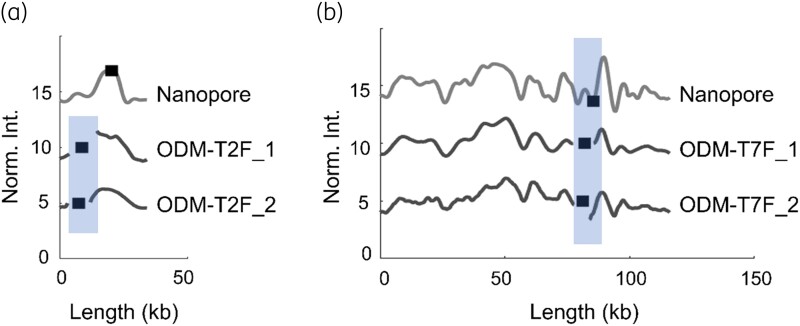
Barcodes of plasmids with the *mcr-1* gene in. (a) Isolates T2F_1 (34 kb) and T2F_2 (34 kb) showing identical plasmids and *mcr-1* gene site in both isolates, compared with the theoretical barcode generated from long-read Nanopore sequencing of T2F_1 (Nanopore). (b) Isolates T7F_1 (116 kb) and T7F_2 (116 kb) showing identical plasmids and *mcr-1* gene site in both isolates, compared with the theoretical barcode generated from long-read Nanopore sequencing of T7F_1 (Nanopore). The *x*-axis shows the length of plasmids in kb and the *y*-axis is the normalized intensity in arbitrary units, where the intensity plots are shifted five units vertically for clarity. Black squares within the shaded regions represent the location of the *mcr-1* gene.

Among the 12 isolates studied, 6 isolates carried the *mcr-1* gene on a ∼34 kb plasmid. Figure 3 compares the ODM and Nanopore barcodes for four of these plasmids (two more in Figure 4(a), discussed below). In all four cases there was a good match (0.05 < *P* < 0.1) between the Nanopore and ODM barcodes. For isolate T1S_1 [Figure 3(a)], the ODM barcode showed a reasonable match (*P* ∼0.1, CC = 0.74), with the *mcr-1* gene location similar to that of the Nanopore barcode. For isolate T4F_2 (Figure 3c), the ODM generated barcode matched well with the Nanopore barcode (*P* < 0.1, CC = 0.93), but there was a shift in the *mcr-1* gene location by ∼15 kb. For isolates T3F_1 and T6F_2, we did not perform Nanopore sequencing, so their ODM barcodes are instead compared with the Nanopore barcode of T1S_1. For T3F_1 (Figure 3b), the barcode matches with the T1S_1 Nanopore barcode (*P* < 0.1, CC = 0.88) but the *mcr-1* gene was shifted by ∼5 kb. On the other hand, the ODM barcode of T6F_2 [Figure 3(d)] had a very good match (*P* < 0.05, CC = 0.93) with the T1S_1 Nanopore barcode with identical *mcr-1* gene location. It is noteworthy that the NGS data showed no *mcr-1* gene for isolate T6F_2, but ODM demonstrated the presence of the *mcr-1* gene in a 34 kb plasmid, the profile of which matched with the Nanopore-generated barcode of isolate T1S_1. ODM in general has been limited to mapping genomic DNA fragments of >50 kb sizes.^43^ Here, we have demonstrated that ODM can be useful in mapping and comparing barcodes of ∼30 kb plasmids and further identify the location of antibiotic resistance genes in the plasmid and compare the results with long-read Nanopore sequencing, but the resolution of the method is limited. Plasmids that are 30–40 kb generally have fewer features in their barcodes due to their shorter length, making the barcodes less unique and we attribute this as the reason for the lower precision in gene location identification in these cases.

Next, we turned our focus to patients with more than one isolate. From the eight patients included in this study, four had two isolates (T2, T4, T6 and T7) collected at different timepoints. Among them, patients T4 (T4F_1: 116 kb; T4F_2: 34 kb) and T6 (T6F_1: 68 kb; T6F_2: 34 kb) carried the *mcr-1* gene on plasmids of different sizes between the two isolates, suggesting that the plasmids were not similar. On the other hand, patients T2 (T2F_1, T2F_2: 34 kb) and T7 (T7F_1, T7F_2: 116 kb) showed identically sized *mcr-1* plasmids in the two isolates. Figure 4(a) shows a comparison between ODM barcodes of isolates T2F_1 and T2F_2 (*P* < 0.01, CC = 0.93), showing similar barcodes and *mcr-1* gene location. When compared with the barcode generated from Nanopore sequencing (Nanopore sequencing of T2F_1), we find that the ODM barcodes match well with the theory (*P* < 0.1, CC > 0.8), but the *mcr-1* gene is at a different location. This again highlights the challenges with smaller plasmids. Figure 4(b) shows that T7F_1 and T7F_2 had identical ODM barcodes (*P*  <  0.01, CC = 0.85) and that the *mcr-1* gene location matched well with the Nanopore barcode of T7F_1.

**Figure 5.**
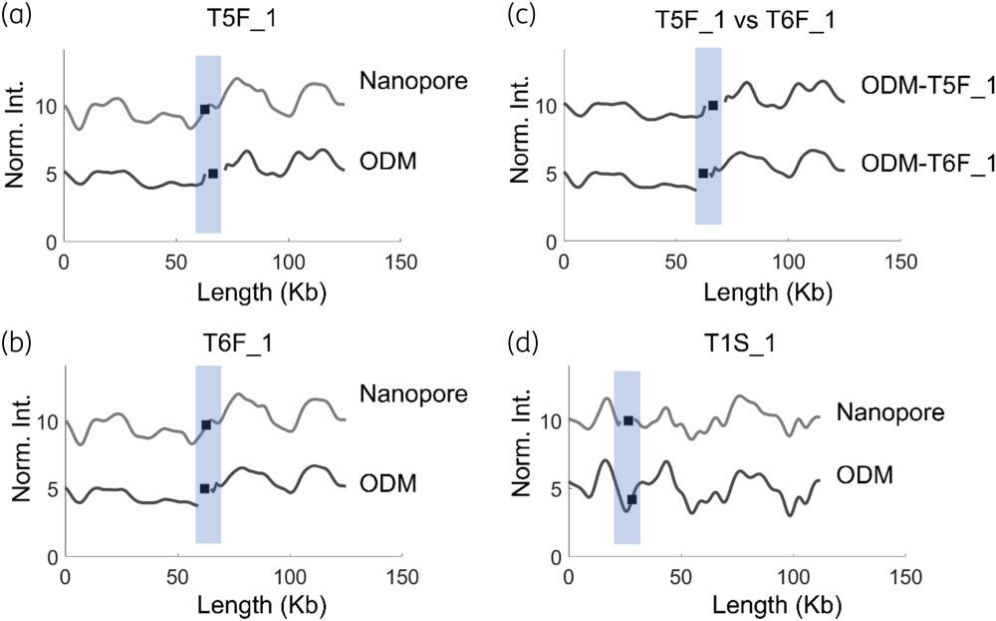
Barcodes of plasmids with other antibiotic resistance genes. The *x*-axis shows the length of the plasmids in kb and the *y*-axis is the normalized intensity in arbitrary units, where the intensity plots are shifted five units vertically for clarity. Black squares in the shaded regions represent the location of the *bla*_CTX-M_ gene. (a) T5F_1 (125 kb plasmid) carrying the *bla*_CTX-M-55_ gene, compared with the Nanopore barcode. (b) T6F_1 (125 kb plasmid) carrying the *bla*_CTX-M-55_ gene, compared with the Nanopore barcode. (c) T5F_1 versus T6F_1, showing identical barcodes and *bla*_CTX-M-55_ gene site. (d) Isolate T1S_1 with a 112 kb plasmid carrying the *bla*_CTX-M-15_ gene, compared with the Nanopore barcode.

ODM has the capability of not only identifying the plasmid with the antibiotic resistance gene, but also giving an overview of the different plasmids present in each isolate. Our ODM experiments targeting the *mcr-1* gene revealed uncut circular plasmids of other sizes in most isolates. Further, the NGS data showed the presence of other resistance genes, mainly *bla*_CTX-M-15_ and *bla*_CTX-M-55_. Therefore, we selected 3 isolates to identify the plasmids carrying the *bla*_CTX-M_ genes. We performed experiments targeting the above two genes and found that isolates T5F_1 [125 kb, Figure 5(a)] and T6F_1 [125 kb, Figure 5(b)] had an identical plasmid (*P* < 0.01) with the *bla*_CTX-M-55_ gene at the same location [Figure 5(c)]. Importantly, this plasmid was not the plasmid carrying the *mcr-1* gene. In isolate T1S_1, a 112 kb plasmid was carrying the *bla*_CTX-M-15_ gene and the result from ODM matched well with the Nanopore barcode [Figure 5(d)], and again the plasmid was not the same as the one carrying the *mcr-1* gene. The experiments targeting the *bla*_CTX-M_ genes also serves as a negative control, where addition of a gRNA targeting the *bla*_CTX-M_ gene only cuts the plasmid that carries that gene and not the plasmid with the *mcr-1* gene. The fact that for both *mcr-1* and *bla*_CTX-M_, the cut only occurs where the gene is present on the plasmid, and not anywhere else, highlights the specificity of the method.

### Cut-and-stretch assay

**Figure 6.**
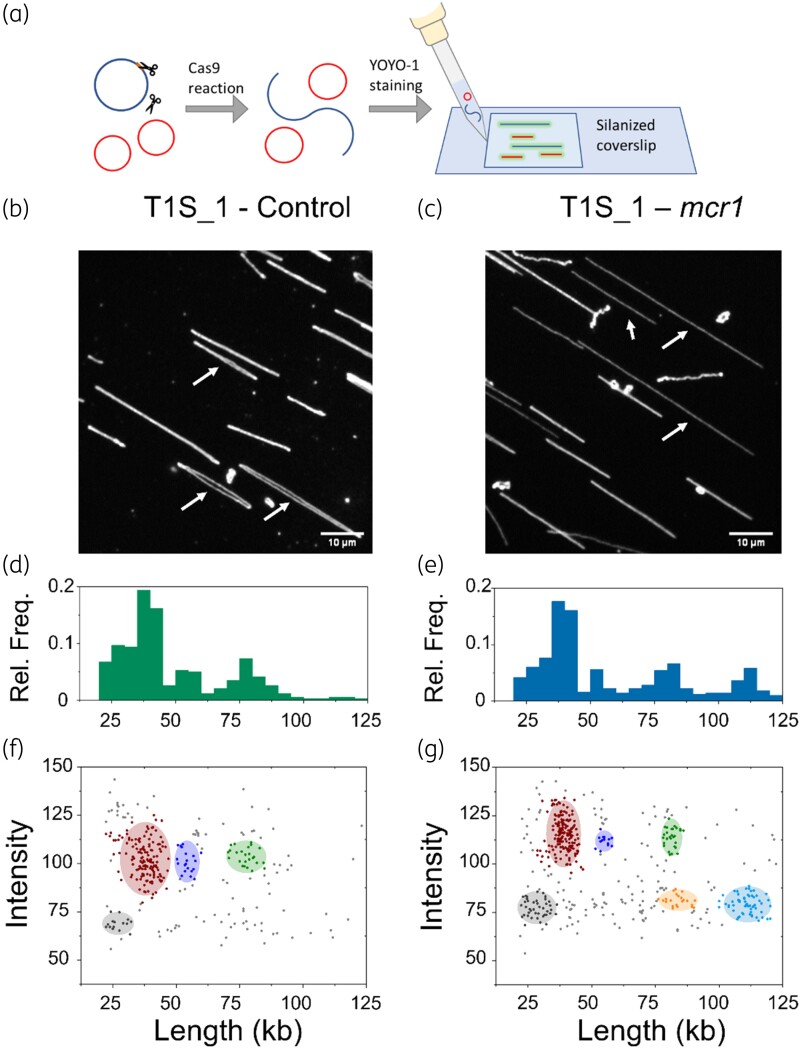
(a) Schematics of the cut-and-stretch microscopy assay. Plasmids isolated from patient samples are treated with CRISPR/Cas9 for targeted restriction of the plasmid with the antibiotic resistance gene of interest. The DNA molecules are then stained with YOYO-1 and stretched on a silanized coverslip for single molecule fluorescence imaging. (b) Fluorescence image from control experiment on plasmids from isolate T1S_1, showing uncut circular plasmids (white arrows). (c) Fluorescence image from experiment using Cas9 targeting the *mcr-1* gene on plasmids from isolate T1S_1, showing linearized plasmids (white arrows). (d–e) Relative frequency versus length plot for the control experiment (d) and for the experiment using Cas9 targeting the *mcr-1* gene (e) for isolate T1S_1. (f–g) Intensity versus length plot for the control experiment (f) and for the experiment using Cas9 targeting the *mcr-1* gene (g) for isolate T1S_1 where coloured regions represent the different clusters identified.

The nanofluidic devices and advanced microscopy setup used for the ODM experiments presented above is not accessible in all laboratories. We therefore recently introduced a simpler assay that identifies if a plasmid carries a certain gene or not [Figure 6(a)]. This assay utilizes the fact that when Cas9 cuts a plasmid the circular plasmid gets linearized.^39^ If the DNA is then stained and stretched, a linearized plasmid will appear twice as long as the same plasmid in its circular form [see Figure 6(b and c)], making it possible to identify a cut by measuring the length of the DNA. Hence, the presence of a specific resistance gene can be confirmed by comparing the length of the DNA present in a plasmid sample when treated with Cas9 and the correct CRISPR gRNA versus a dummy gRNA. Additionally, since stretched circular plasmids consist of two overlapping DNA strands the intensity from a circular plasmid will be higher than from a linearized plasmid, which consists of one DNA strand. To measure the length and intensity of the DNA, it is stained with YOYO-1 after the Cas9 reaction, stretched on a glass surface coated with silanes, and observed using a microscope with basic functionalities (Zeiss Primo Star iLED).

In the control sample, treated with dummy gRNA, four clusters were found at 27, 38, 54 and 78 kb [Figure 6(d)]. Among these four clusters, three (38, 54 and 78 kb) were observed at a high emission intensity, indicating that these are circular plasmids (plasmids 1, 2 and 3, respectively) of approximately double the size [Figure 6(f)]. The cluster at 27 kb with a lower intensity was assigned to fragmented DNA present in the sample.

In the sample treated with gRNA for the *mcr-1* gene, we identified four clusters at 29, 40, 55 and 81 kb, matching well with the control sample [Figure 6(e)]. In addition to this, two new clusters were identified at 84 and 111 kb, but at emission intensities lower than for the circular plasmid clusters, suggesting that these two clusters constitute linear DNA. The lengths of these DNA molecules are approximately the same as the length of plasmid 1 and 2, respectively (2 × 40 = 80 kb and 2 × 55 = 110 kb) indicating that these new clusters correspond to linearization of those plasmids [Figure 6(g)].

Linearization of a plasmid can either be due to Cas9 treatment or random double-strand breaks during sample preparation. By comparing the number of DNA molecules found in the circular versus the linear form for each plasmid we found that only 12% of the plasmid 1 molecules were linearized, while 82% of plasmid 2 molecules were. Figure 6(f) shows that also in the control there is some random linearization of plasmid 1. From this we conclude that only plasmid 2 (111 kb) is linearized due to Cas9 treatment and hence carries the *mcr-1* gene. This agrees with the ODM analysis and Nanopore sequencing of the T1S_1 sample where the *mcr-1* gene was on a 116 kb plasmid [see Figure 2(a)]. The small fraction of linear DNA identified at 84 kb is likely from random double-strand breaks during sample preparation.

### Conclusions

In summary, we have demonstrated the usefulness of ODM in mapping plasmids carrying the antibiotic resistance gene *mcr-1*. We compared our ODM barcodes with barcodes generated from long-read Nanopore sequencing and proved that ODM could provide long-range sequence information and identify the plasmid harbouring the *mcr-1* gene in all the isolates studied. Further, earlier studies on *mcr-1*-harbouring plasmids in *E. coli* isolated from humans and food sources have shown that conjugative plasmids of type IncX4 of sizes 33–35 kb are among one of the two most common plasmid types in plasmid-mediated global spread of colistin resistance.^11,15^ Long-read sequencing techniques, such as Nanopore sequencing, PacBio sequencing and SMRT sequencing are capable of read lengths in the 10–30 kb range,^44,45^ whereas optical mapping-based techniques have been promoted as a tool to map DNA molecules of >100 kb lengths.^46^ We have here pushed the application of ODM to map relatively small plasmids of sizes ∼30 kb, bridging the gap between them. In this work, we consistently identified the location of the *mcr-1* gene in all six isolates with short plasmids. Further, comparison with barcodes from long-read Nanopore sequencing showed a ‘good match’ for all six isolates.

We also identified two patients (T2 and T7) where isolates from samples collected at different times show identical plasmids, suggesting that ODM can be used to study how long a patient is colonized for.^37^ Interestingly, two other patients (T4 and T6) showed different plasmids between isolates from samples collected at different times.

The ODM mapping is applicable to any gene of interest, and in this study we also identified plasmids containing the genes *bla*_CTX-M-15_ and *bla*_CTX-M-55_. In none of the samples studied did we find the *mcr-1* and *bla*_CTX-M_ genes on the same plasmid. This type of information can be of high interest from an epidemiological perspective, for example to understand potential co-selection of resistance.

Finally, we have also demonstrated the possibility of identifying *mcr-1*-encoding plasmids using microscopes that are available in low- and middle-income countries using a simplified cut-and-stretch assay. As a proof-of-concept, we showed the presence of the *mcr-1* gene on a 111 kb plasmid.

It is important to also note that the assay can be directly applied to any other *mcr* gene of interest by designing a gRNA that targets that gene, making the assay even more widely applicable. In addition, the method can be used in primary clinical samples. Our method has been successfully applied to study clinical faecal samples^47^ and identification of bacterial species from uncultured clinical urine samples.^48^

## Supplementary Material

dlad004_Supplementary_Data

## Supplementary data

Supplementary Methods and Table S1 are available as Supplementary data at *JAC-AMR* Online.
