# Supplementary material for: Identification and characterization of plasmids carrying the mobile colistin resistance gene mcr-1 using optical DNA mapping
Source: JAC Antimicrob Resist. 2023 Feb 1;5(1):dlad004. doi: 10.1093/jacamr/dlad004 (PMC9891347; doi:10.1093/jacamr/dlad004)
Supplement: dlad004_Supplementary_Data [file dlad004_supplementary_data.docx]

**Supporting information**

**Identification and characterization of plasmids carrying the mobile colistin resistance gene *mcr-1* using optical DNA mapping**

Sriram KK^1^, Moa S. WRANNE^1^, Tsegaye Sewunet^2^, Elina Ekedahl^1^, Maarten Coorens^3^, Teerawit Tangkoskul^4,^ Visanu Thamlikitkul^4^, Christian G. Giske^2,3^, Fredrik Westerlund^1*^

*^1^Department of Biology and Biological Engineering, Chalmers University of Technology, Gothenburg, Sweden.*

*^2^Division of Clinical Microbiology, Department of Laboratory Medicine, Karolinska Institute, Stockholm, Sweden.*

*^3^Clinical Microbiology, Karolinska University Hospital, Stockholm, Sweden*

*^4^Faculty of Medicine Siriraj Hospital, Mahidol University, Bangkok, Thailand*

*Corresponding author. Email: [*fredrikw@chalmers.se*](mailto:fredrikw@chalmers.se)

**Running title: Optical DNA mapping of plasmids carrying mcr-1**

**Materials and Methods:**

**1. DNA extraction and genome sequencing**

Two to three fresh pure colonies of E. coli and K. pneumoniae were taken from culture grown overnight and suspended in 200 µL suspension buffer from the EZ1®DNATissue Kit (QIAGEN). Then, DNA was extracted using an EZ1 Advanced DNA Bacteria Card on the EZ1 Advanced extraction system. Concentration of the extracted DNA was measured using Qubit™ 3.0 (Waltham, MA, USA). NEXTRA-XT kits were used for library preparation and sequencing was performed on an Illumina HiSeq2500 microbial sequencing platform.

The raw reads were assembled using SPAdes 3.13.1 and the assembled draft genomes were used to query resistome and virulome profile of the isolates from publicly available databases such as ResFinder (https://cge.cbs.dtu.dk/services/ResFinder/), and Comprehensive Antimicrobial Resistance Database (CARD) (https://card.mcmaster.ca/analyze/rgi). The draft genomes of the K. pneumoniae isolates were also used for in silico prediction of both the capsular loci (KL) and O-lipopolysaccharide by using the Kaptive/Holt laboratory database at (https://kaptive-web.erc.monash.edu/init/default/jobs). Furthermore, all the isolates were sequenced on the MinIOn Nanopore long read sequencing platform using the 1D Native barcoding genomic DNA (with EXP-NBD104, EXPNBD114, and SQK-LSK109) barcoding and ligation kit. Both short-read and long-read sequences were submitted to the NCBI, (BioProject ID: PRJNA850449).

Plasmids were extracted using a NucleoBond® Xtra Midi Kit (Macherey-Nagel) plasmid purification protocol. Bacterial strains were inoculated into 100 mL selective medium (Luria–Bertani with 30 µg/mL ampicillin) and incubated overnight at 37 °C, followed by DNA extraction using NucleoBond Xtra Midi Kit (Macherey-Nagel). Each isolate was pelleted by centrifugation at 7000 rpm, for 10 min at 4 °C. The pellet was resuspended in resuspension buffer, lysed, and purified on columns according to the manufacturer’s recommendations. The eluted plasmid DNA was precipitated with isopropanol and washed once with 70% ethanol and dried at ambient temperature. The dried pellet was reconstituted in 100 μL TE buffer. The DNA concentration was measured using a Qubit™ dsDNA BR Assay Kit (ThermoFisher Waltham, MA, USA).

**2. Optical DNA mapping (ODM)**

The ODM protocol involving CRISPR/Cas9 based restriction of plasmids harboring the antibiotic resistance gene of target, followed by one-step competitive labelling of DNA molecules using YOYO-1 and netropsin, to visualize DNA molecules at the single molecule level in nanochannels, using fluorescence imaging has been described in detail in earlier studies ^1, 2^.

First, the guide RNA (gRNA) was formed by mixing equimolar amounts of CRISPR RNAs (crRNA with sequence 5´TATGCGTAAGCCACGCCTAG3´ for the mcr-1 gene, 5´CCGTCGCGATGTATTAGCGT3´ for the blaCTX-M genes, 0.5 nmol, Dharmacon Inc.) and trans-activating crRNAs (tracrRNA, 0.5 nmol, Dharmacon Inc.) in 1X NEB-3 buffer (New England Biolabs) and 1X bovine serum albumin (BSA, 0.1 µg/ml). 10 mM RNAse-free Tris-EDTA buffer (Sigma-Aldrich) was added to adjust the total volume of the solution to 15 µl. This mixture was incubated at 4 °C for 30 min.

600 ng of Cas9 protein (PNA Bio Inc.) was then incubated with the gRNA from the step above, at 37°C for 15 min. Further, 60 ng of plasmid DNA was mixed with the solution containing Cas9-gRNA complex and incubated at 37°C for 1h, to enable targeted restriction and linearization of the plasmid with the gene of interest. Next, equal amounts of plasmid DNA and λ-DNA for internal size reference (48,502 bp, New England Biolabs) were incubated with YOYO-1 (1:2 bp molar ratio, Invitrogen) and netropsin (60:1 bp molar ratio, Sigma-Aldrich) in 0.5X Tris-borate-EDTA buffer (TBE, Sigma-Aldrich) at 50 ºC for 30 min, forming the ODM barcode ^3, 4^.

Fluorescence single molecule imaging of the above prepared sample in nanochannel devices was carried out using an inverted fluorescence microscope (Zeiss AxioObserver.Z1) equipped with a 100x oil immersion objective (Zeiss, NA = 1.46), an FITC filter (488 nm excitation / 530 nm emission), and an EMCCD camera (Photometrix Evolve). Fabrication scheme for the nanochannel devices using lithography and other micro- nanofluidic fabrication processes has been discussed in detail elsewhere ^5, 6^.

Image analysis was carried out using custom-written MATLAB codes and the details of the statistical methods used in the analysis are discussed in detail elsewhere ^7^. Briefly, images of 50 frames, with 100 milliseconds exposure time for each frame were recorded using an EMCCD camera. These images were then converted to multi-TIFF files, from which DNA barcodes were obtained for each DNA molecule. Since we have many nanochannels in parallel, it is easy to obtain barcodes of tens of DNA molecules from a single image. These barcodes are then used to obtain a consensus intensity profile (Figure 1-iii) and barcodes from plasmid isolates of different patients are compared against each other or against a nanopore sequencing generated theoretical barcode. A statistical test is performed to obtain p-values, which are then used to compare barcodes for similarity, with p < 0.01 being a “good match” and p > 0.01 being “no match”. For the 34 kb plasmids, p-values were observed to be 0.05 < P < 0.1, as minor differences between the barcodes in comparison results in higher p-values, due to the short lengths. To validate our results for the 34 kb plasmids, we additionally looked into the Pearson correlation coefficient (CC-value) of the compared barcodes. Briefly, CC-value is a measure of similarity between two barcodes in comparison, with CC-value of -1 meaning no similarity and CC-value of 1 meaning very high similarity. For the 34 kb plasmid barcodes, in addition to the p-value being 0.05 < P < 0.1, we used a cut-off of CC-value > 0.8 suggesting “good match”. The details on estimating CC-values are discussed in detail in our earlier work ^7^.

**3. Cut and stretch assay**

Experiments were performed using Primo Star iLED (Zeiss) equipped with Reflected-light fluorescence illuminator iLED 455 nm for excitation, a 485 nm long pass emission filter, a Plan-Achromat 100x oil immersion objective (Zeiss, NA = 1.25) and a mobile phone camera (Samsung S20). For this assay, DNA molecules were immobilized on coverslips, prepared using a protocol described elsewhere. (37) The DNA sample was diluted to a concentration of 0.13 µM in 0.05X TBE buffer with 2 % (v/v) of β-Mercaptoethanol. 3.4 µl of the DNA sample was then added at the edge of a functionalized coverslip placed on top of a 76 mm × 26 mm microscope slide (Menzel-Gläser, Thermo Scientific).

For Cas9 mediated restriction of antibiotic resistance gene in plasmids, the same protocol discussed under ODM (Materials and Methods, section 2.1) was used. The only difference here is that the fluorescence staining was done with YOYO-1 at 3:1 bp molar ratio and no netropsin was used. Experiments were done using plasmids from patient T1 (T1S_1), targeting the mcr-1 gene. For the control experiment, a dummy gRNA with no sequence complementarity in sample T1S_1 was used (5′ GGTCCTTGTAACCATCGGTG 3′).

For imaging, the main camera of a Samsung S20 was used. Images were acquired in jpeg and dng file formats with the following settings F-stop: f/1.8, Exposure time: 1 second, ISO speed: ISO-200. The .dng files were used for analysis. Using ImageJ, the blue and red channels were discarded before pixels were binned 2x2 and the images smoothed and saved as tiff-files. Images were then treated as described in detail in our earlier work ^8^. The following settings were used: threshold = -7, width = 1 –10 pixels, length = 60 – ∞ pixels, minimum eccentricity = 0.54 – 0.74, minimum molecule-to-convex-hull ratio = 0.54 – 0.74. Length and average intensity of the molecules were retrieved as described. To convert from µm to kb, separate measurements were done with λ-DNA (48 502 bp). λ-DNA was measured in triplicate and found to have an average length of 20.4 ± 0.8 µm.

For clustering DNA molecules into groups in the intensity vs length plot (Figures 6e and 6f) MATLABs built-in algorithm “DBSCAN” was used. The neighborhood search radius was set to 5 and 6.4 for sample and control respectively and the minimum numbers of neighbors required to identify a core point was set to 11 and 13. Prior to clustering, the intensity was normalized to approximately the same span as the length – to make both equally important in clustering.

**Table 1: Susceptibility to β-lactam antimicrobials based on disk diffusion, colistin (MIC), and resistome of the isolates and the results from ODM.**

Key: S - Susceptible, I – Intermediate, R – Resistant

Note: All isolates were positive for mcr-1 gene using PCR.

| **Isolate No.** | **Isolate ID** | **Species ID** | **Antibiotics tested** | | | | | **B-lactamase and colistin resistance genes** | **Results from ODM** |
| --- | --- | --- | --- | --- | --- | --- | --- | --- | --- |
|  |  |  | **Ceftriaxone** | **Ceftazidime** | **Cefepime** | **Ertapenem** | **Colistin (MIC)** |  |  |
| T1S_1 | CTR-020-S01 | *K. pneumoniae* | R | R | R | S | R(8) | *bla*_CTX-M-15_, mcr-1 | mcr-1 identified in 34 kb plasmid and *bla*_CTX-M-15_ in 112 kb plasmid |
| T2F_1 | CTR-030-F02 | *E. coli* | R | R | R | S | R(4) | *bla*_CTX-M-55,_ mcr-1 | mcr-1 identified in 34 kb plasmid |
| T2F_2 | CTR-030-F03 | *E. coli* | R | R | R | S | R(4) | *bla*_CTX-M-55,_ mcr-1 | mcr-1 identified in 34 kb plasmid |
| T3F_1 | CTR-031-F04 | *E. coli* | R | R | R | S | R(16) | *bla*_CTX-M-55,_ mcr-1 | mcr-1 identified in 34 kb plasmid |
| T4F_1 | CTR-033-F02 | *K. pneumoniae* | R | R | R | R | R(64) | *bla*_CTX-M-14,_ *bla*_NDM-1_ mcr-1 | mcr-1 identified in 116 kb plasmid |
| T4F_2 | CTR-033-F03 | *E. coli* | R | R | R | S | R(4) | *bla*_CTX-M-55_, mcr-1 | mcr-1 identified in 34 kb plasmid |
| T5F_1 | CTR-036-F04 | *E. coli* | R | R | R | R | R(4) | *bla*_CTX-M-55,_ mcr-1 | mcr-1 identified in 68 kb plasmid and *bla*_CTX-M-15_ in 125 kb plasmid |
| T6F_1 | CTR-037-F06 | *E. coli* | R | R | R | S | R(8) | *bla*_CTX-M-55_, mcr-1 | mcr-1 identified in 68 kb plasmid and *bla*_CTX-M-15_ in 125 kb plasmid |
| T6F_2 | CTR-037-F07 | *E. coli* | R | R | R | S | R(32) | *bla*_SHV-12,_ No mcr-1 gene | mcr-1 identified in 34 kb plasmid |
| T7F_1 | CTR-074-F04 | *E. coli* | R | S | I | S | R(8) | *bla*_CTX-M-15_, *bla*_CTX-M-14,_ *bla*_NDM-1,_mcr-1 | mcr-1 identified in 116 kb plasmid |
| T7F_2 | CTR-074-F05 | *E. coli* | R | I | R | S | R(16) | *bla*_CTX-M-14_, bla_CTX-M-55_, *bla*_OXA-232,_ mcr-1 | mcr-1 identified in 116 kb plasmid |
| T8X_1 | C008/5 | *E. coli* | - | R | - | R | R(8) | *bla*_CTX-M-15_, *bla*_CTX-M-14,_ *bla*_OXA-232,_ mcr-1 | mcr-1 identified in 68 kb plasmid |

**References:**

1. Kk S, Ekedahl E, Hoang NTB et al. High diversity of bla(NDM-1)-encoding plasmids in Klebsiella pneumoniae isolated from neonates in a Vietnamese hospital. *Int J Antimicrob Agents* 2021: 106496.

2. Müller V, Rajer F, Frykholm K et al. Direct identification of antibiotic resistance genes on single plasmid molecules using CRISPR/Cas9 in combination with optical DNA mapping. *Scientific Reports* 2016; **6**: 37938.

3. Nilsson AN, Emilsson G, Nyberg LK et al. Competitive binding-based optical DNA mapping for fast identification of bacteria - multi-ligand transfer matrix theory and experimental applications on Escherichia coli. *Nucleic Acids Research* 2014; **42**: e118-e.

4. Nyberg LK, Persson F, Berg J et al. A single-step competitive binding assay for mapping of single DNA molecules. *Biochemical and Biophysical Research Communications* 2012; **417**: 404-8.

5. Kk S, Lin YL, Sewunet T et al. A Parallelized Nanofluidic Device for High-Throughput Optical DNA Mapping of Bacterial Plasmids. *Micromachines (Basel)* 2021; **12**.

6. Persson F, Tegenfeldt JO. DNA in nanochannels—directly visualizing genomic information. *Chemical Society Reviews* 2010; **39**: 985.

7. Dvirnas A, Pichler C, Stewart CL et al. Facilitated sequence assembly using densely labeled optical DNA barcodes: A combinatorial auction approach. *PLOS ONE* 2018; **13**: e0193900.

8. Goyal G, Ekedahl E, Nyblom M et al. A simple cut and stretch assay to detect antimicrobial resistance genes on bacterial plasmids by single-molecule fluorescence microscopy. *Scientific Reports* 2022; **12**.
